# Case Report: IgG4-RD-related ophthalmopathy combined with monoclonal gammopathy of undetermined significance

**DOI:** 10.3389/fimmu.2025.1565388

**Published:** 2025-05-12

**Authors:** Shanshan Liang, Wei Xu, Peiying Zhong, Chuanmin Tao, Li Zhang, Chengyao Jia

**Affiliations:** ^1^ Department of Laboratory Medicine, West China Hospital, Sichuan University, Chengdu, China; ^2^ Department of Clinical Laboratory, The Affiliated Taian City Central Hospital of Qingdao University, Taian, China; ^3^ Department of Laboratory, 363 Hospital, Chengdu, China; ^4^ Department of hematology, West China hospital, Sichuan University, Chengdu, China

**Keywords:** IgG4-RD-related ophthalmopathy, diagnostic criteria, M protein, IgE, monoclonal gammaglobulinemia of undetermined significance

## Abstract

IgG4-related disease (IgG4-RD), an immune-mediated fibroinflammatory disorder, has few reports in combination with monoclonal gammopathy of undetermined significance (MGUS). Herein, we present a case of a 69-year-old woman with manifestations of left orbital occupation and visual acuity decline. Ancillary tests indicated persistent positivity of IgG4 antibody, and IgG4-RD-related ophthalmopathy was diagnosed based on the criteria. Concurrently, serum protein electrophoresis revealed an M protein level of 12.23 g/L. Immunofixation electrophoresis suggested a positive IgG λ-type M protein, and MGUS was diagnosed in conjunction with bone marrow smear and flow cytometry.

## Introduction

IgG4-related disease (IgG4-RD), an immune-mediated fibroinflammatory disorder, typically has a chronic insidious or subacute onset and can affect multiple organs and systems throughout the body. The most common clinical manifestations include a significantly elevated serum IgG4 concentration and mass-like lesions. Monoclonal gammopathy of undetermined significance (MGUS) is characterized by the clonal proliferation of plasma cells or B cells that secrete monoclonal immunoglobulin. However, it does not reach a sufficient load to cause organ damage and is considered a benign precursor disease (1). Herein, we report the management of a patient with combined IgG4-RD ophthalmopathy and MGUS.

## Case description

A 68-year-old female patient was admitted to the hospital due to “persistent redness and swelling of the left eye for 9 months and visual impairment for half a year”. Since the disease onset, the left eye had become red without an obvious cause, accompanied by eyelid swelling, photophobia, tearing, a foreign body sensation, vision loss, and diplopia. She was initially prescribed oral prednisone at a dose of 30mg once a day. Although the eye swelling improved slightly, the eye remained red, with persistent tearing and an obvious foreign body sensation, and the vision did not recover. One month ago, she revisited the ophthalmology outpatient clinic, where she was advised to discontinue prednisone and underwent orbital MRI scanning with enhancement. The results showed a protruding left eyeball, thickened left extraocular muscles, and a blurred orbital fat gap, with a slight protrusion of the left eyeball, suggesting the possibility of inflammatory lesions.

**Figure 1:**
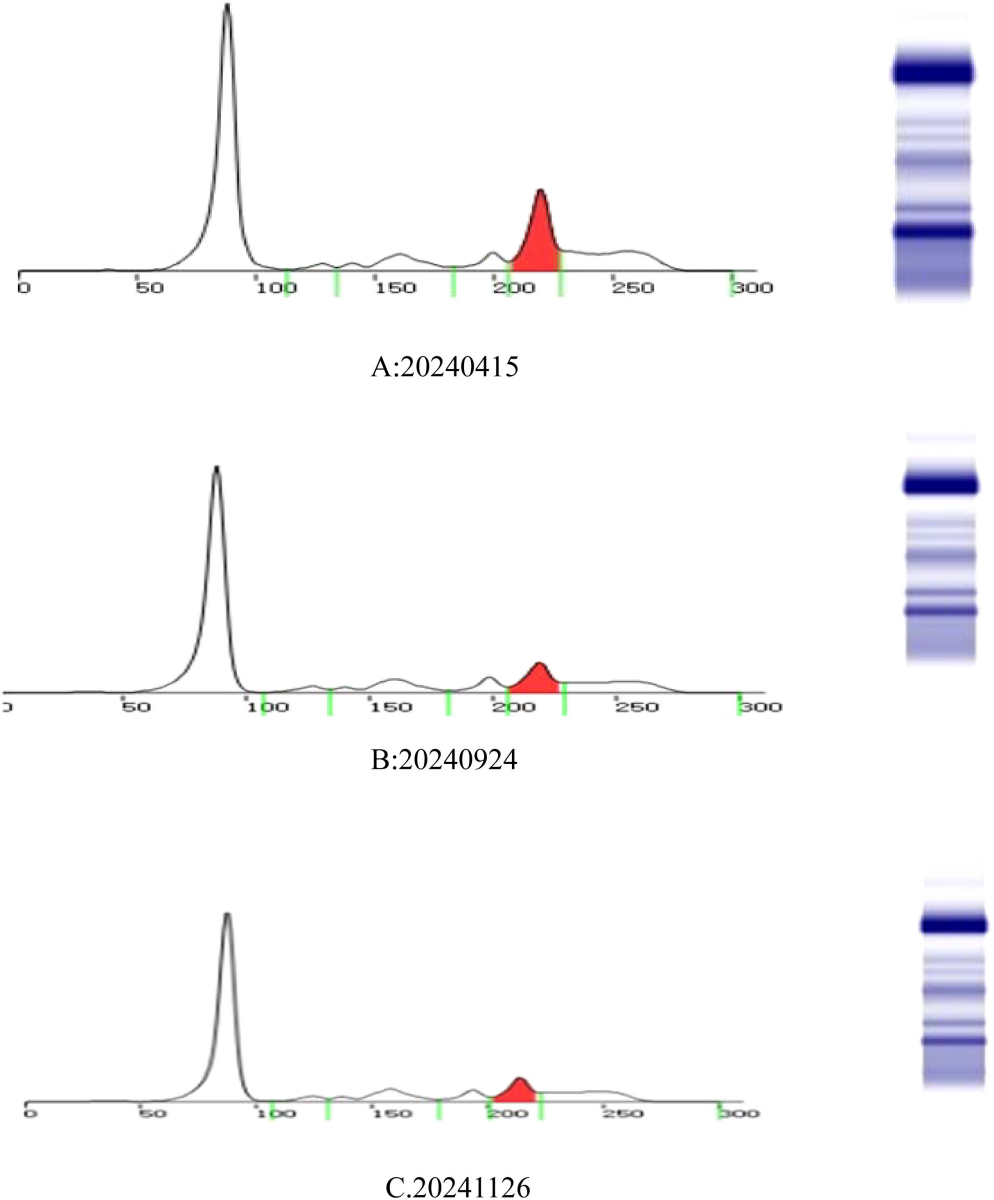
Serum protein electrophoresis: M protein colored by red. The three testing dates are 15 April 2024 **(A)**, 24 September 2024 **(B)**, and 26 November 2024 **(C)**.

**Figure 2:**
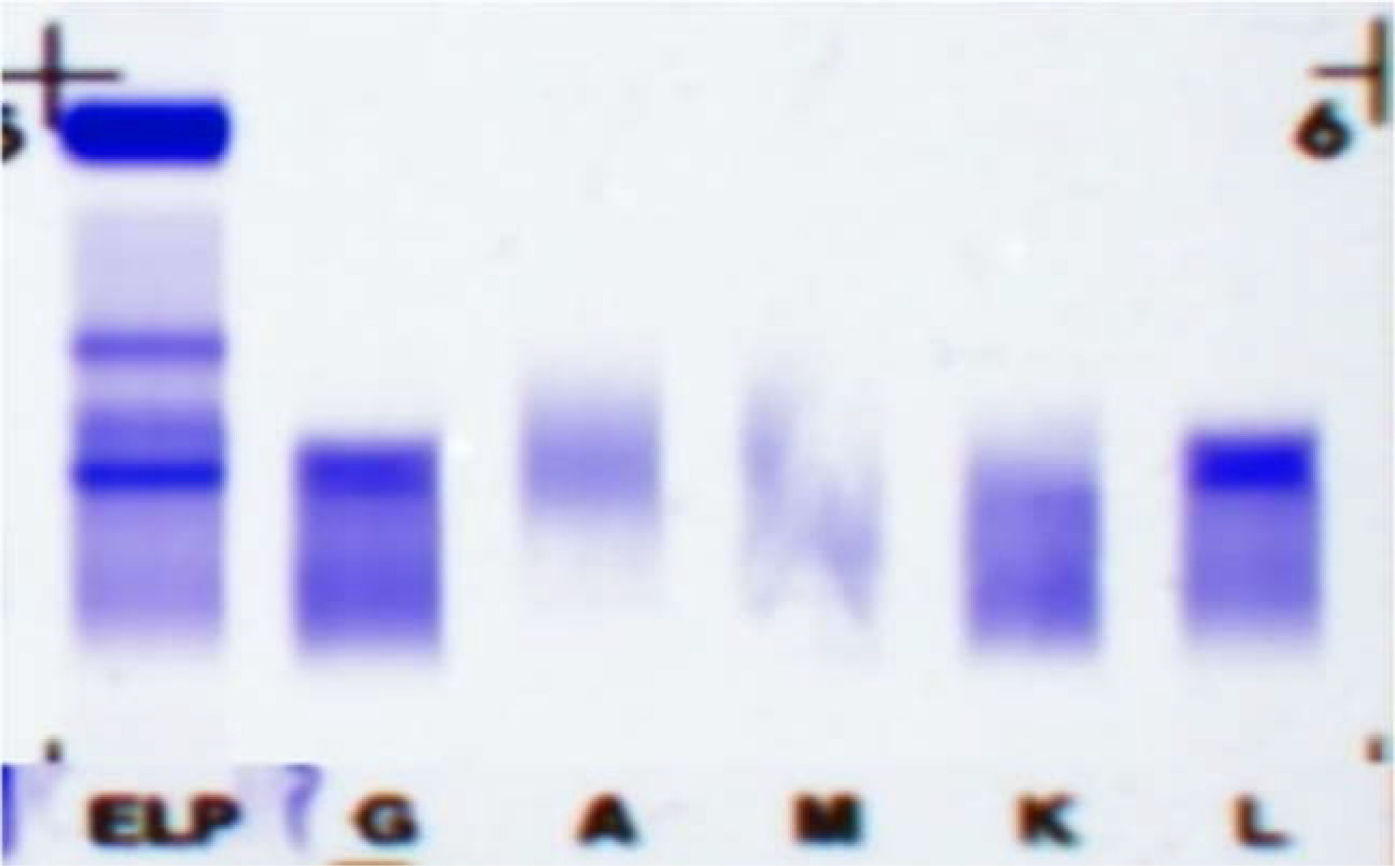
Immunofixation electrophoresis: M protein was of IgG λ type.

**Figure 3:**
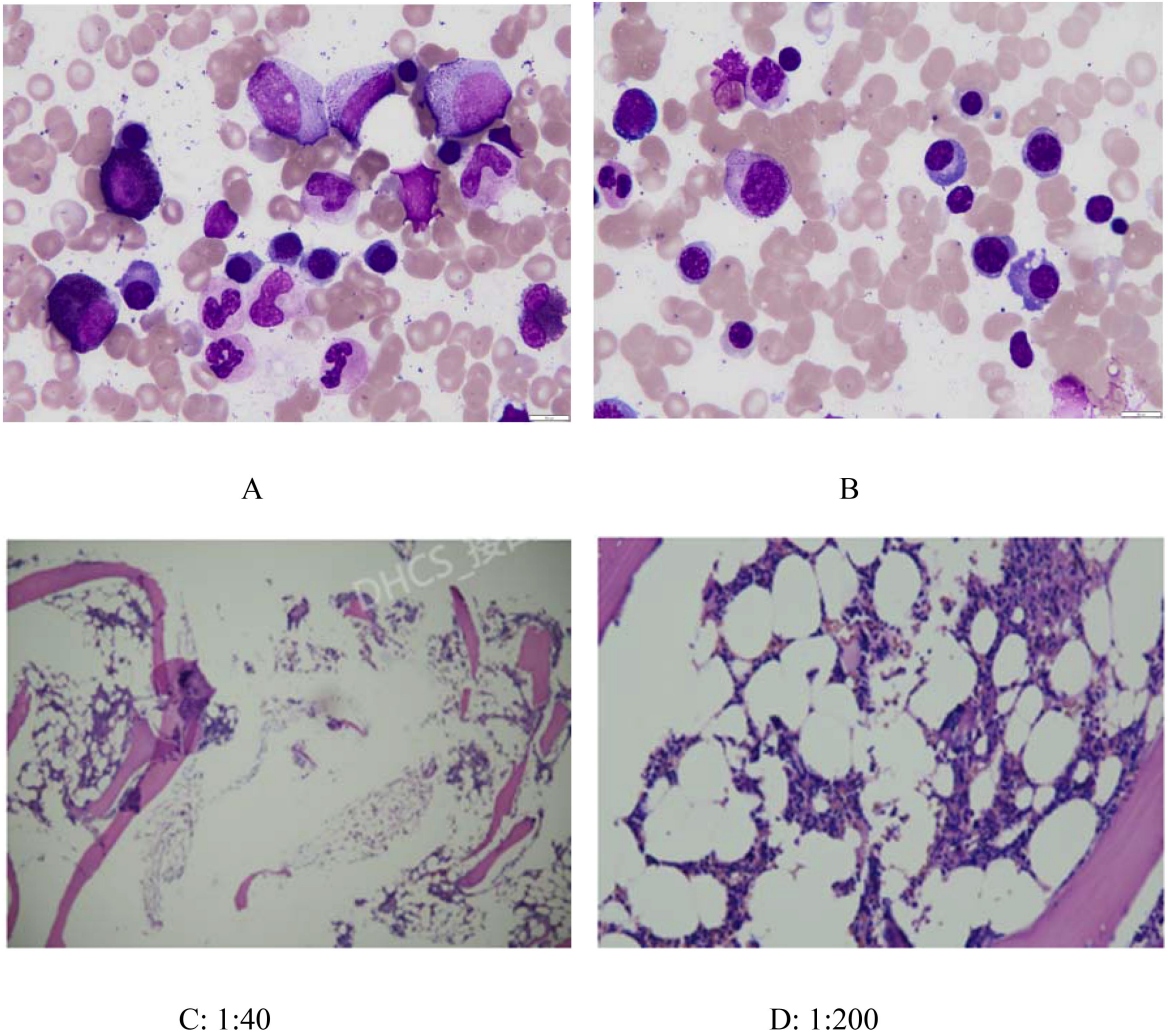
Bone marrow smear cytology(AB) and Bone marrow biopsy(CD). **(A)** and **(B)**: Bone marrow was actively proliferative, with a slightly higher red lineage in 40.5% of cases and a low percentage of plasma cells in 1%. **(C)** (1:40) and **(D)** (1:200): Histopathology: Minimal bone cortex and serous exudate observed. Hematopoietic-to-fat tissue ratio approximately 1:2–3. Myeloid-to-erythroid ratio approximately 3–4:1, predominantly segmented neutrophils (MPO+). Megakaryocytes: 2–3 per HPF. No significant morphological abnormalities observed in the three hematopoietic lineages (myeloid, erythroid, megakaryocytic). Lymphocytes and plasma cells show focal or scattered distribution. Reticular fiber staining (FOOT): Reticular fibers are not increased (MF-0).

**Figure 4:**
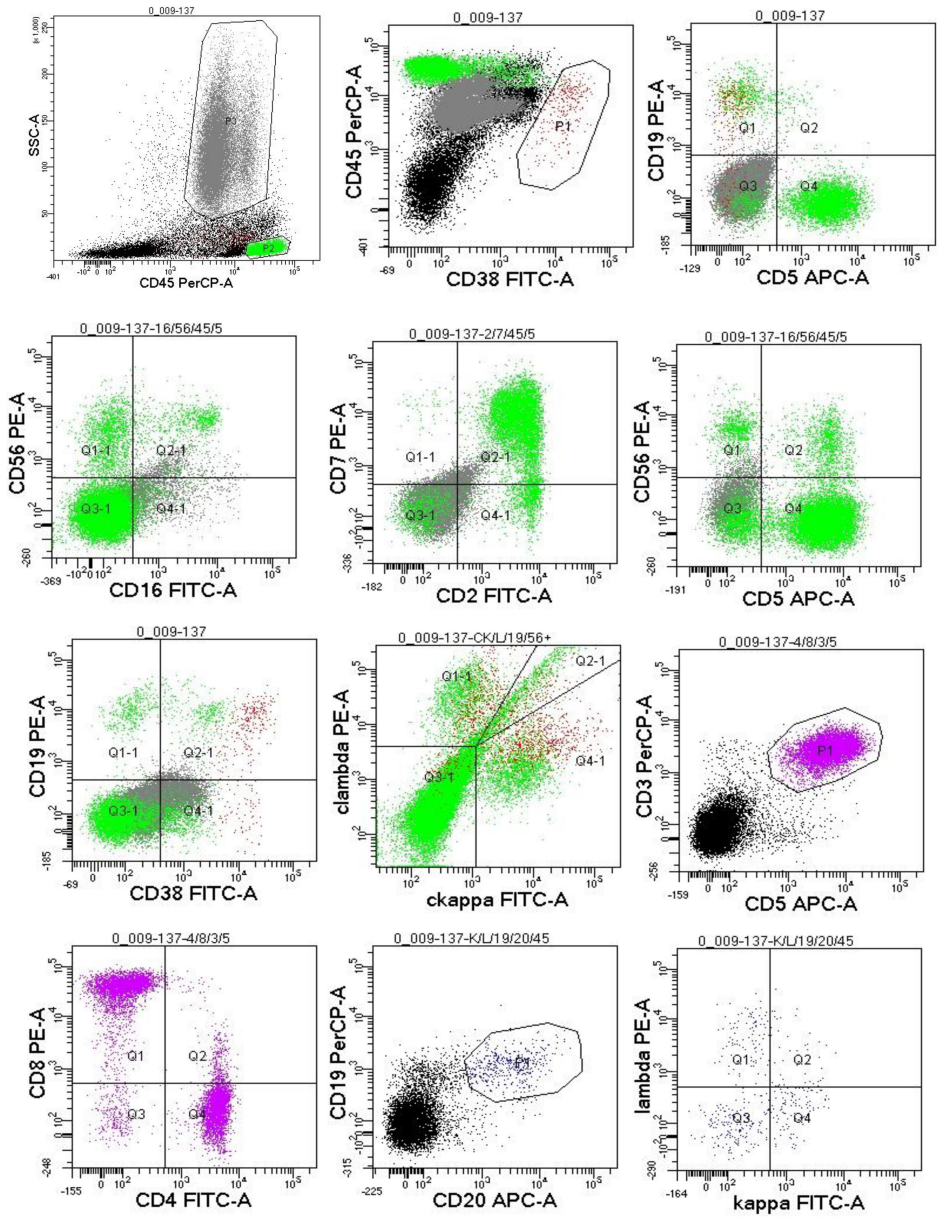
Flow cytometric immunophenotyping: no obvious abnormal phenotypical cell clusters were seen.

**Figure 5:**
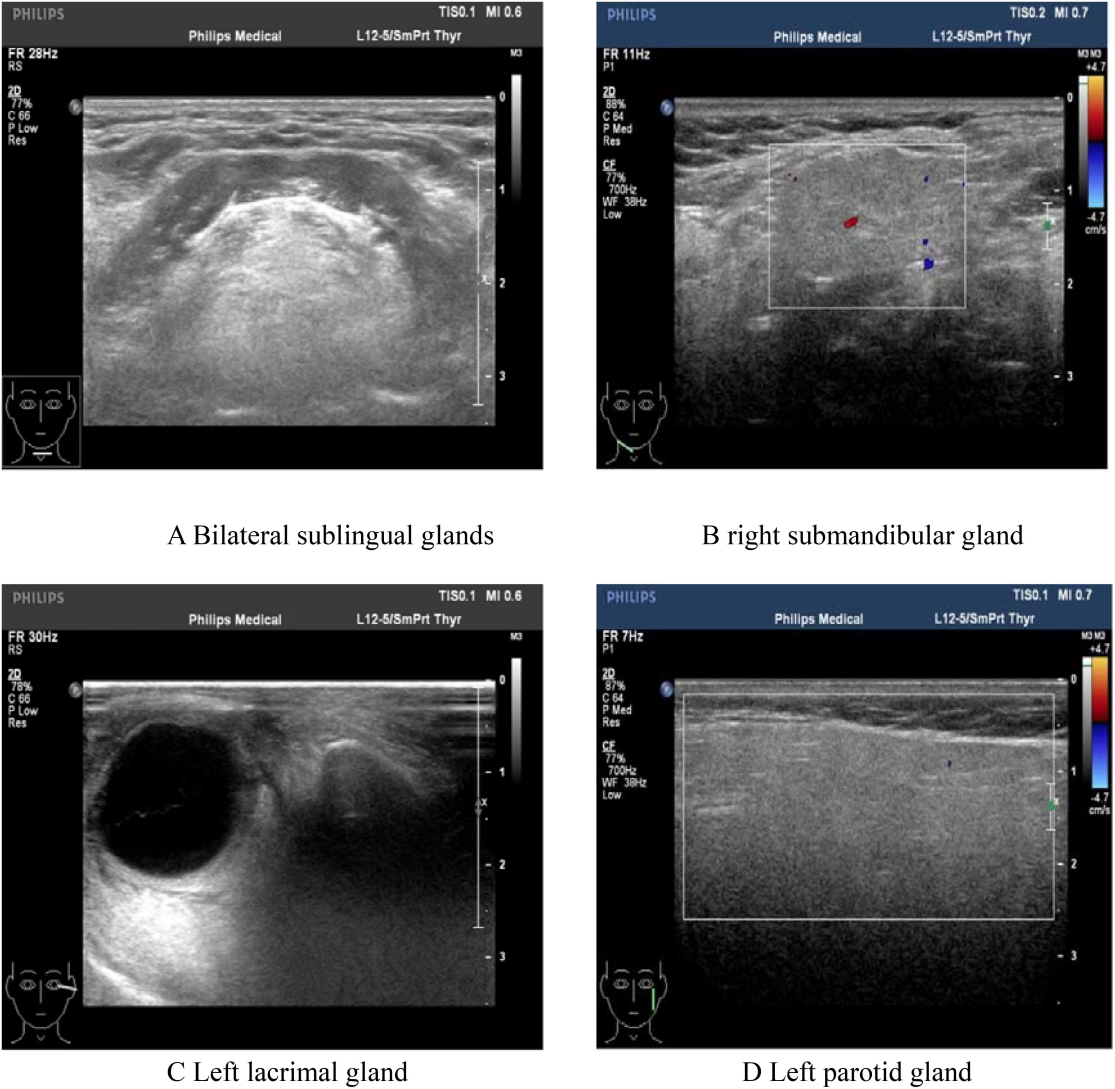
Color Doppler ultrasonography showed: heterogeneous changes in the bilateral lacrimal glands and submandibular glands. **(A)** Bilateral sublingual glands; **(B)** Right submandibular gland; **(C)** Left lacrimal gland. **(D)** Left parotid gland.

**Figure 6:**
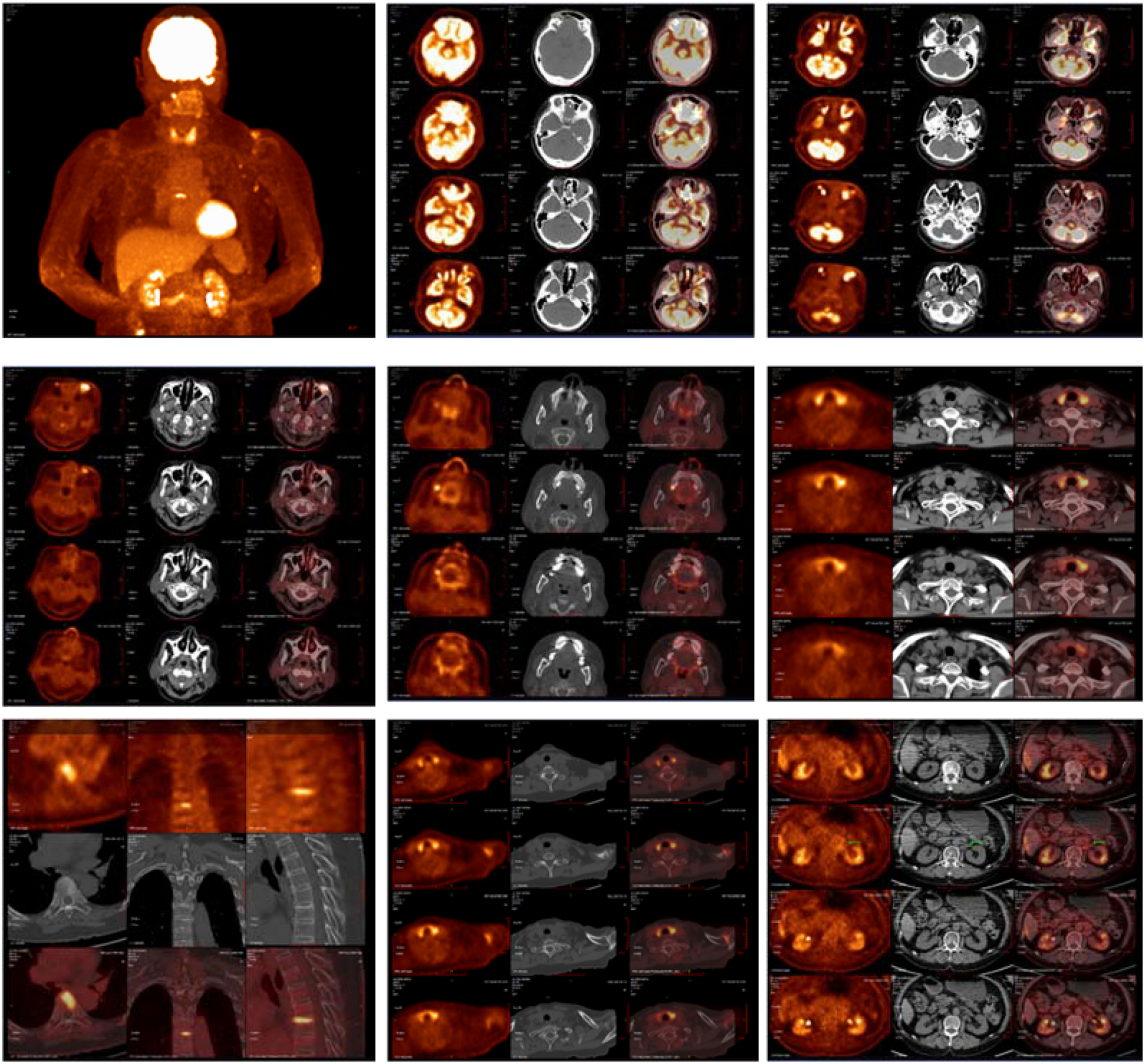
The imaging findings of PET-CT: 1. Abnormally increased glucose metabolism in the left orbital soft tissue and thyroid lesions, suggestive of IgG4-related disease involvement. 2. Glucose metabolism elevation in the T7 upper endplate lesion and para-aortic soft tissue at the left renal level: Etiology? IgG4-related disease involvement cannot be excluded; clinical correlation is recommended. 3. Left maxillary sinusitis; right upper alveolar periodontitis/periarthritis; left acromioclavicular joint lesion, likely inflammatory. 4. Left axillary lymph node lesions, likely inflammatory or reactive changes.

Since the onset of the disease, blood tests showed immunoglobulin G4 subtype 16.2 g/L (reference interval 0.03-2.01 g/L), immunoglobulin G 19.9 g/L (reference interval 8.6-17.4 g/L), total immunoglobulin E 379 IU/ml (reference interval <165 IU/ml), and red blood cell count 3.34×10 ^12^/L (reference interval 3.8-5.1×10^12^/L), hemoglobin 108 g/L (reference interval 115–150 g/L), sedimentation rate 88 mm/h (reference interval <38 mm/h), total protein 71.1 g/L, albumin 41.8 g/L, globulin 29.3 g/L. Calcium was 2.17 mmol/L, urea was 7.9 mmol/L, creatinine was 64 μmol/L, and there was no abnormality in liver and kidney function, blood glucose and blood lipids, and thyroid hormone examination. Serum protein electrophoresis showed M protein (12.23g/L) (**Figure 1A**), and immunofixation electrophoresis showed that M protein was of IgG λ type (**Figure 2**). κ light chain test 8.52 g/L (reference interval 6.29-13.5 g/L), λ light chain test 21.8 g/L (reference interval 3.13-7.23g/L), B-cell fine subpopulation: plasmacytoma cell subpopulation count 4.270cell/ul (reference interval 0–4 cell/ul). Bone marrow smear cytology (**Figure 3A, B**): Bone marrow was actively proliferative, with a slightly higher red lineage in 40.5% of cases and a low percentage of plasma cells in 1%. Bone marrow biopsy (**Figures 3C, D**): Hypocellular bone marrow with decreased hematopoiesis (age-related), showing hypoplasia of all three lineages. Intracellular and extracellular iron staining: extracellular iron 2+, ferritic granulocytes 0.28, type I ferritic granulocytes 0.16, type II ferritic granulocytes 0.12. Flow cytometric immunophenotyping (**Figure 4**): no obvious abnormal phenotypical cell clusters were seen. Ultrasound showed (**Figure 5**): heterogeneous changes in the lateral lacrimal and submandibular glands. The patient’s current condition made it difficult to obtain a biopsy specimen. PET/CT head and neck imaging differential diagnosis (**Figure 6**): left orbital soft tissue and thyroid lesions with abnormally elevated glucose metabolism, with a tendency to IgG4-related disease involvement; T7 upper endplate lesions and left renal level para-abdominal aortic soft tissue with increased glucose metabolism, with no exclusion of IgG4-related disease involvement; left maxillary sinusitis; and right alveolar periodontitis/periarthritis. The left acromioclavicular joint lesions were mostly inflammatory, and the left axillary lymph node lesions tended to be inflammatory or reactive changes. According to the 2019 ACR/EULAR IgG4-related disease diagnostic criteria (2), the patient was diagnosed with “IgG4-RD-related ophthalmopathy and monoclonal gammopathy of undetermined significance”. Hormone therapy was initiated. The follow-up plan was as follows: (1) For IgG4-RD-related ophthalmopathy, the patient was scheduled to be followed up in the outpatient clinic of the Department of Rheumatology and Immunology one month after hospital discharge to adjust the treatment plan. (2) For MGUS, it was recommended that the patient follow up in the outpatient clinic of the Department of Hematology every three months and undergo reviews of blood routine, electrolytes, liver and kidney functions, and serum protein electrophoresis.

**Figure 7:**
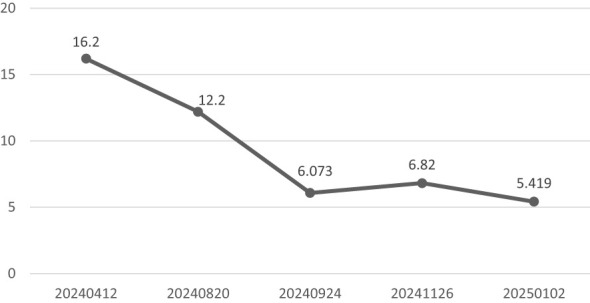
Longitudinal Trends in Serum IgG4 Levels During Clinical Follow-Up.

During longitudinal follow-up documented in the Hospital Information System (HIS), the ocular symptoms of the patient showed resolution and received the following protocol-based therapeutic regimen. Prednisone acetate tablets (5 mg × 100 tablets): 1) Initial phase: 20 mg once daily for 15 days. 2)Dose reduction: 17.5 mg once daily for 30 days. 3)Maintenance phase: 15 mg once daily for an additional 30 days, followed by 15 mg once daily for 28 days. Concurrently, thalidomide tablets (50 mg× 20 capsules) were administered at a stable dose of 50 mg nightly throughout sequential treatment phases (15 days, 30 days, 30 days, and 28 days). Serial laboratory monitoring demonstrated a 50.7% reduction in serum M protein levels (**Figure 1**): Baseline: 12.23 g/L (15 April 2024); Post-induction: 6.39 g/L (24 September 2024); Final follow-up: 6.03 g/L (26 November 2024) (Total observation period: 225 days). IgG4 levels remained above the upper limit of normal (2.010 g/L) throughout the 9-month monitoring period, with a 66.6% decline from the peak value (16.200 g/L to 5.419 g/L)(**Figure 7**). This pattern is consistent with IgG4-related disease (IgG4-RD) activity, though histopathological confirmation is required for definitive diagnosis.

## Discussion

IgG4-related disease (IgG4-RD) frequently presents with polyclonal hypergammaglobulinemia, and approximately one-third of patients have an associated allergic disease with increased IgE levels (3). In this case, the total IgE level was 379 IU/ml (reference value: <165 IU/ml). It was observed that the plasmablast subpopulation counts were elevated, and plasmablasts were notably increased in the peripheral blood of patients with IgG4-associated disease. These plasmablasts secreted greater amounts of IgG4, and their number was positively correlated with the serum IgG4 level, the number of involved organs, and the degree of disease activity.

This case demonstrates the clinical utility of the 2019 ACR/EULAR IgG4-related disease (IgG4-RD) classification criteria in a patient with multiorgan involvement and diagnostic complexities. The patient achieved a total inclusion score of 33 points(Threshold: ≥20 points), fulfilling the criteria through a combination of elevated serum IgG4, bilateral glandular involvement, and retroperitoneal lesions. Below, we contextualize the scoring process within broader diagnostic and therapeutic considerations. 1. Entry Criteria. The patient met entry criteria through characteristic involvement of the orbit (proptosis, extraocular muscle thickening), lacrimal/submandibular glands (heterogeneous ultrasound changes), retroperitoneum (aortic soft tissue lesion), and thyroid (diffuse FDG uptake). These findings align with the criteria’s emphasis on organ-specific patterns. 2. Exclusion Criteria. No exclusion criteria were met, critical for maintaining specificity: 1)Clinical: Partial response to glucocorticoids (30 mg/day) did not meet the threshold for “no objective response” (≥40 mg/day for 4 weeks). 2)Serologic/Radiologic: Absence of ANCA positivity, eosinophilia, or features suggesting malignancy/infection (e.g., necrosis, rapid progression). 3)Pathologic: While biopsy was not performed, imaging lacked granulomatous or necrotizing features atypical for IgG4-RD. 3 Inclusion Criteria Scoring: 1) Serum IgG4 (11 points): A markedly elevated IgG4 level (16.2 g/L, >5× ULN) provided strong diagnostic weight. Extreme IgG4 elevations correlate with multiorgan disease and higher relapse risk. 2)Gland Involvement (14 points): Bilateral lacrimal and submandibular gland lesions (≥2 sets) scored maximally, reflecting the criteria’s prioritization of typical organ patterns. 3)Retroperitoneum (8 points): The infrarenal aortic soft tissue lesion matched the criteria’s definition of “circumferential/anterolateral periaortic involvement,” a hallmark of IgG4-RD. 4) Other Domains: Chest, pancreas, kidney, and pathology domains contributed no points due to absent findings or unavailable biopsy.

IgG4-RD can affect multiple organs and tissues throughout the body, exhibiting a wide range of onset symptoms and clinical manifestations. The most commonly involved tissues/organs include lymph nodes, submandibular gland, lacrimal gland, and pancreas, while others involve the lungs, bile ducts, sinuses, parotid glands, retroperitoneal tissues, aorta, kidneys, skin, thyroid, pituitary gland, dura mater/dura, pericardium, and mediastinum (4). In this particular case, the main involvement was the lacrimal gland, presenting as unilateral or bilateral painless enlargement. The patient might experience a foreign body sensation and ocular discomfort. Unfortunately, due to the specific location, a biopsy diagnosis could not be performed.

Owing to the anatomical structure, the involvement of ocular structures is difficult to ascertain by physical examination alone and requires clarification with the aid of imaging. Imaging may reveal bilateral diffuse symmetrical enlargement of the lacrimal glands or enlargement of the involved tissues with mass infiltration, such as enlargement of the extraocular muscles surrounded by diffuse and heterogeneous masses, and may be accompanied by other immune disorders. PET/CT scans for SUV elevations in the Submandibular glands: No abnormal ^18^F-FDG uptake; Maxillary sinuses:Left: Mucosal thickening on CT, but no significant uptake (SUV max 1.8). Right: Normal (SUV max 1.6); Lymph nodes: Cervical: No uptake (SUV max < 2.0). Left axillary: Mild uptake (SUV max 3.68), but nodes were small (<10 mm) with preserved morphology. These findings suggest no active IgG4-RD involvement in the submandibular glands or maxillary sinuses. Mild axillary node uptake likely reflects reactive changes. Peri-Orbital Region (Left): Markedly increased ^18^F-FDG uptake (SUV max 8.15), correlating with clinical ocular symptoms and supporting IgG4-related ophthalmic disease (IgG4-ROD). Elevated serological IgG4 (≥ 1.35 g/L) is a crucial serological feature for considering this disease and is highly indicative of it. It has been documented that patients with IgG4-RD have significantly higher mean serum IgG4 levels and an elevated IgG4/IgG ratio. The area under the curve (AUC) for the diagnostic validity of the serum IgG4/IgG ratio for IgG4-RD was 0.921 (95% CI, 0.876 - 0.965) compared with healthy individuals (5). In this case, the calculated IgG4/IgG result was 0.805, which holds clinical significance for diagnosis.

Monoclonal gammopathy of undetermined significance (MGUS) is a condition where plasma cells or B cells undergo clonal proliferation, consequently secreting monoclonal immunoglobulins. However, they do not reach a certain load and do not cause organ damage (6). It is regarded as a benign precursor disease that may progress to lymphoproliferative disorders or multiple myeloma. The prevalence of MGUS rises with age (7). Most MGUS patients are over 65 years of age (8) and most do not progress to overt diseases. However, in a small number of cases, multiple comorbidities may occur, including an elevated risk of fractures, renal impairment, peripheral neuropathy, secondary immunodeficiency, and cardiovascular diseases (9). In the literature, a case of IgG4-associated submandibular gland involvement accompanied by an increase in monoclonal immunoglobulin has been reported (10). Additionally, ocular manifestations of MGUS have also been reported (11–14), However, no reports of IgG4-related eye disease combined with MGUS were retrieved.

The patient’s IgG λ M-protein (12.23 g/L) and elevated λ free light chains (21.8 g/L) raise questions about the relationship between IgG4-RD and monoclonal gammopathy. While MGUS is classically associated with plasma cell dyscrasias, growing evidence suggests it may also arise secondary to chronic inflammatory or autoimmune conditions (15). In IgG4-RD, persistent antigenic stimulation and dysregulated B-cell proliferation—hallmarks of the disease—could drive clonal plasma cell expansion, leading to monoclonal gammopathy (16). Notably, elevated serum globulin levels (29.3 g/L in this case) and polyclonal hypergammaglobulinemia, common in IgG4-RD, may further predispose to MGUS by fostering a pro-inflammatory microenvironment. This patient’s multiorgan involvement and markedly elevated IgG4 (16.2 g/L) align with prior observations that MGUS in IgG4-RD correlates with extensive disease and higher IgG4 levels. Chronic inflammation, driven by cytokines such as IL-6 and BAFF, may promote both polyclonal and monoclonal plasma cell responses. Importantly, MGUS in IgG4-RD rarely progresses to myeloma but requires monitoring, as clonal evolution has been reported in rare cases. As reported by Tanaka (17), intracranial lesions of IgG4-RD accompanied by smoldering multiple myeloma (MM) are highly uncommon and must be distinguished from plasmacytoma and other conditions. In this patient, plasmacytoma was ruled out based on the polyclonality of plasma cells and the absence of an increase in serum monoclonal protein and myeloma cells in the bone marrow. Consequently, the diagnosis was IgG4-RD. the concurrent presence of MM complicated the diagnosis of IgG4-RD.

The observed partial response to corticosteroids with adjunctive thalidomide aligns with reports of combined immunomodulatory therapy in refractory IgG4-RD. While glucocorticoids remain first-line, relapse rates exceed 50% during tapering, necessitating steroid-sparing agents (18–20). Thalidomide, a TNF-α inhibitor, may suppress B-cell hyperactivity and fibrosis in IgG4-RD, synergizing with corticosteroids. Notably, the 50.7% decline in M-protein suggests thalidomide’s dual role in mitigating both IgG4-RD activity and clonal plasma cell expansion, as seen in inflammatory MGUS.

As emphasized by Culver et al. (21), elevated serum IgE (defined as >125 kIU/L) is observed in 54% of IgG4-RD patients, a prevalence significantly higher than healthy controls (16%, *P* < 0.0001) and disease controls with elevated IgG4 from other conditions. This finding aligns with prior retrospective studies (34–86% IgE elevation in IgG4-RD subsets) and underscores that IgE elevation is a common, though not universal, feature of IgG4-RD. Similar findings have been reported in studies by Hu J.Q (22). and Yazici B (23). Critically, Culver et al. demonstrated that serum IgE >480 kIU/L distinguishes IgG4-RD from mimickers with 86% specificity and a likelihood ratio of 3.2, supporting its utility in differential diagnosis. Beyond diagnosis, IgE serves as a marker for disease relapse: IgE >380 kIU/L at baseline predicts relapse with 88% specificity and 64% sensitivity, identifying patients who may require closer monitoring. Mechanistically, IgE elevation in IgG4-RD correlates with peripheral eosinophilia (38% of patients, *P* = 0.004) and tissue infiltration by IgE-positive mast cells, suggesting an IgE-mediated allergic component in this disease. These observations link IgG4-RD to Th2-driven immune responses, a key pathophysiological pathway also implicated in its fibroinflammatory process. While IgG4 remains the most specific serologic marker, integrating IgE measurement offers additive value, particularly in cases with atypical IgG4 levels (e.g., 9% of IgG4-RD patients have normal IgG4 at diagnosis, of whom only 1 had elevated IgE). This aligns with current guidelines advocating a multimodal approach to IgG4-RD diagnosis, combining clinical, serologic, radiologic, and pathologic data. In summary, elevations in IgE are common in IgG4-RD (affecting over half of patients) and represent a valuable biomarker for both diagnosis and relapse prediction, as robustly demonstrated by Culver et al.’s prospective cohort.

Both IgG4-RD and MGUS can involve multiple organ systems. Historically, IgG4-RD has been considered to predominantly present with polyclonal immunoglobulin (Ig) elevation; thus, it is critical to identify the origin of monoclonal M protein during the diagnostic workup. The diagnostic and therapeutic approach in this case offers valuable insights for the clinical management of such conditions.

## Data Availability

The original contributions presented in the study are included in the article/supplementary material. Further inquiries can be directed to the corresponding authors.
